# Mysteriously Puffy Extremities: An Unintended Consequence of Intravenous Drug Abuse

**DOI:** 10.7759/cureus.25453

**Published:** 2022-05-29

**Authors:** Abhishek Janardan, Malek Ayoub, Husna Khan, Pinky Jha, Mohan S Dhariwal

**Affiliations:** 1 Internal Medicine, Medical College of Wisconsin, Milwaukee, USA; 2 Internal Medicine, Medical College of Wisconsin, Wauwatosa, USA; 3 Dentistry, Midwestern University Chicago College of Osteopathic Medicine, Chicago, USA

**Keywords:** drug abuse, lymphatic obstruction, edema of hand, illicit drugs, puffy extremities

## Abstract

Puffy hand syndrome is a rare manifestation due to continuous intravenous drug abuse. It is a form of lymphedema caused by the sclerosing nature of intravenously administered drugs. It typically presents with bilateral, non-pitting edema at the dorsum of the hands. Proper identification of puffy hand syndrome represents a crucial junction of interest to physicians as the syndrome can be used to recognize a patient’s past or ongoing drug addiction. Here, we present the case of a homeless 27-year-old presenting with erythema and edema in his extremities.

## Introduction

Dr. Hans Abeles, the Commissioner of Health for the Department of Corrections in New York City, initially described puffy hand syndrome in 1965 [1]. Puffy hand syndrome is an uncommon clinical phenomenon associated with repeated intravenous drug abuse. It typically presents with contour changes at the dorsum of the hands, which can extend proximally to the forearm [2]. Common risk factors for puffy hand syndrome include female gender, injection in hands, and poor antiseptic technique [3]. It affects between 7% and 16% of intravenous drug users [4]. However, the frequency and severity of the complications of this syndrome are rarely reported. Here, we present the case of a homeless 27-year-old presenting with asymmetric edema in his extremities.

## Case presentation

**Figure 1:**
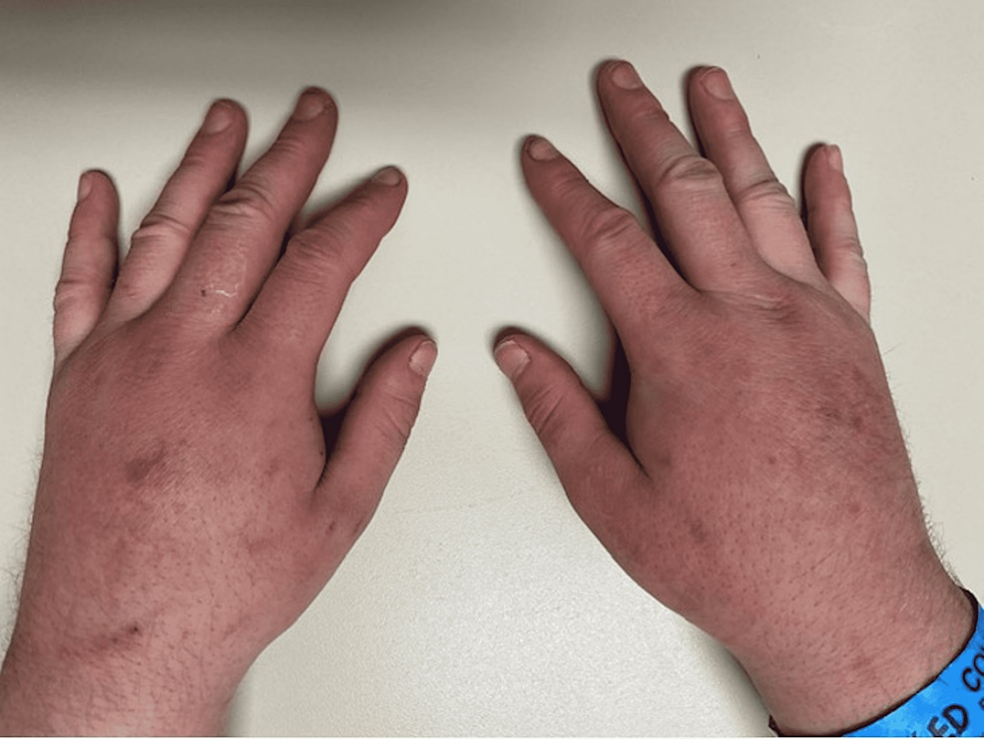
Edema with erythema on the dorsum of the hands.

**Figure 2:**
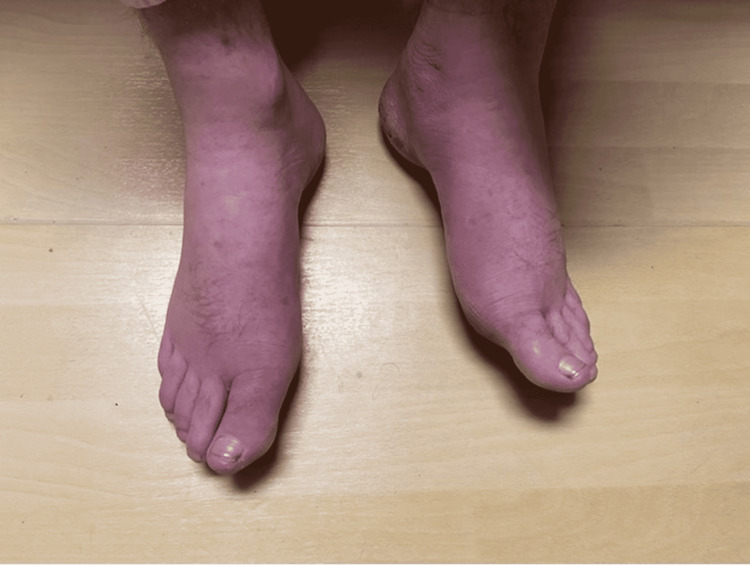
Edema in the feet. The left foot displays a mid-arch punctate area.

A 27-year-old, homeless, Caucasian male presented to an urgent care with erythema and edema in his extremities and a small abscess with surrounding cellulitis on his right hand. He reported routinely injecting heroin. The patient completed a course of ceftriaxone and a two-week course of amoxicillin/clavulanate, which yielded moderate improvement in the swelling. However, the swelling never completely dissipated. The patient then presented at the emergency department six months later with edema and erythema overlying his extremities (Figures 1, 2).

He stated that he injected heroin the morning of his admission and smoked crack cocaine the day before he was admitted. He denied fever, chills, shortness of breath, pleuritic chest pain, orthopnea, dyspnea on exertion, or any urinary symptoms. He reported progressive swelling of both upper extremities and left lower extremity a month prior to his presentation. A large lump on the anterior aspect of his right arm was also noted, notably around sites of heroin injection. The patient did not notice any drainage from any injection site. He mentioned that the swelling began asymmetrically, bilaterally in the legs, and progressed to the right and left upper extremities. The patient also reported using new needles and injecting heroin in all four extremities.

On presentation, the patient was afebrile and saturated 98% on room air. Physical examination demonstrated warm extremities with normal capillary refill and strong bilateral distal pulses. Pitting edema was noted in various locations. Pitting edema in the right hand was seen with volar erythema. Edema in the right foot and left-hand edema displayed a punctate area around the mid-arch with associated tenderness to palpation without overlying redness, crepitus, or fluctuance. Blood cell count revealed mild leukocytosis to 12.0 × 10^9^/L. The comprehensive metabolic panel was unremarkable. A 2/6 systolic murmur was appreciated throughout the precordium. Multiple linear fibrotic scars were observed along the forearms and the antecubital fossa. The urine drug screen was positive for benzodiazepines, tetrahydrocannabinol (THC), cocaine, and opiates. Human immunodeficiency virus was negative, coronavirus disease 2019 was negative, and blood cultures were negative. Histoplasma/Blastomyces urine antigens were negative. Urinalysis did not display proteinuria, and transaminases were within normal limits. Ultrasound showed occlusive cephalic vein thrombosis in the right upper extremities, without any evidence of thrombosis in the lower extremities. The infectious disease team was consulted for further evaluation.

Initially, cellulitis was suspected due to edema and erythema, mild leukocytosis, and neutrophilia. The patient was started on cefazolin 1 g for 10 days. Infectious disease deemed the lesions to be inconsistent with vasculitis associated with endocarditis. Cefazolin was discontinued. The patient was diagnosed with puffy hand syndrome.

## Discussion

Puffy hand syndrome can present during or after bouts of intravenous drug abuse with intermittent non-pitting edema [3]. These clinical symptoms are typically found on the dorsum of the hands [4]. After several months of evolution, the edema does not decrease, even with postural changes [3]. Foot involvement is less common yet remains possible if injections are frequently administered in the surrounding area. In addition, infectious complications such as cellulitis regularly support the diagnosis. Unfortunately, these clinical findings are commonly misdiagnosed and persist for years before a correct diagnosis is made. Some implicated risk factors for puffy hand syndrome include female gender, repeated injections in the hands and feet, and the absence of tourniquet use [5]. Although this disease primarily impacts chronic drug users, a speculated cause for its underdiagnosis is the underutilization of medical care due to feelings of guilt and shame [2].

The pathophysiology of puffy hand syndrome is multifaceted but poorly understood [4]. Both venous and lymphatic involvement is speculated. It is believed to be a form of lymphedema that arises due to the sclerosing nature of intravenously administered drugs. It is thought to occur due to lymphatic blockage that originates from the direct toxicity of the injected drug, drainage of impurities, or other infectious complications [3]. Lymphatic destruction and obstruction have also been theorized as modes of pathogenesis for puffy hand syndrome [5].

The diagnosis of puffy hand syndrome is one of exclusion; laboratory findings tend to be ambiguous. Clinically, puffy hand syndrome is particularly noted with edema of the hands and feet. The associated swelling can mask the associated tendons and veins on the dorsum of the hands and feet [5]. This finding is noted in the pictures of our patients’ extremities. The edema has also been observed to originate sporadically and asymmetrically, increasing the probability of becoming permanent and symmetrical with continued intravenous drug abuse.

The differential diagnosis for puffy hand syndrome typically includes lymphangitis, venous thrombosis, heart failure, renal insufficiency, liver failure, erythromelalgia, scleroderma, rheumatoid arthritis, and systemic lupus erythematosus (SLE) [6]. The generalized presentation factors of SLE include fatigue, fever, lymphadenopathy, and weight loss. Skin manifestations include malar rash or photosensitive rashes that can last for several weeks after sun exposure. Musculoskeletal findings associated with SLE include generalized arthralgia that can be accompanied by morning stiffness [7]. Rheumatoid arthritis is a chronic inflammatory disease that primarily affects joints, resulting in deformities and loss of function. Extra-articular presentations of rheumatoid arthritis can also include fever, generalized weight loss, pericardial inflammation, vasculitis, and compression neuropathy [8]. This patient’s edematous findings were initially a manifestation of cellulitis associated with infective endocarditis. However, this diagnosis was ruled out due to blood cultures being negative. Additionally, our patient’s presentation involving puffed-up extremities with a history of intravenous drug abuse separated it from autoimmune illnesses such as SLE and rheumatoid arthritis as many of the systemic and organ-specific symptom criteria for both diseases were not met.

Treatment regimens for puffy hand syndrome are non-specific. In general, prevention of skin infections and cold environments can be considered. Therapeutic options for symptom relief tend to decrease the increased hydrostatic pressure within affected vessels. Common options include compression garments, lymphedema physical therapy, and lymphatic massage.

## Conclusions

Puffy hand syndrome is defined by symmetric, painless erythema and edema of the hands in patients with a history of intravenous drug use. It is a rare complication that carries the potential of being confused with systemic sclerosis, rheumatoid arthritis, or erythromelalgia. Injection practices are likely to cause puffy hands, warranting the necessity of educational campaigns directed toward patients who struggle with intravenous drug use. Given this condition’s recognition in the addiction medicine and vascular surgery realms and the rising rates of drug abuse within the United States, general practitioners should improve their awareness of this complication to ensure proper and timely treatment.
